# Phylogeny and Disease Association of Shiga Toxin–producing *Escherichia coli* O91

**DOI:** 10.3201/eid1509.090161

**Published:** 2009-09

**Authors:** Alexander Mellmann, Angelika Fruth, Alexander W. Friedrich, Lothar H. Wieler, Dag Harmsen, Dirk Werber, Barbara Middendorf, Martina Bielaszewska, Helge Karch

**Affiliations:** Institute of Hygiene and the National Consulting Laboratory on Hemolytic Uremic Syndrome, Münster, Germany (A. Mellman, A.W. Friedrich, D. Harmsen, B. Middendorf, M. Bielaszewska, H. Karch); Department of Periodontolgy, Münster (D. Harmsen); Robert Koch Institute, Wernigerode, Germany (A. Fruth); Free University Berlin, Berlin, Germany (L.H. Wieler); Robert Koch Institute, Berlin (D. Weber)

**Keywords:** Shiga toxin, Escherichia coli O91, STEC, MLST, hemolytic uremic syndrome, subtyping, disease association, bacteria, enteric diseases, dispatch

## Abstract

The diversity and relatedness of 100 Shiga toxin–producing *Escherichia coli* O91 isolates from different patients were examined by multilocus sequence typing. We identified 10 specific sequence types (ST) and 4 distinct clonal groups. ST442 was significantly associated with hemolytic uremic syndrome.

Shiga toxin–producing *Escherichia coli* (STEC) infections are public health concerns because of the severe illnesses they cause, such as hemorrhagic colitis and hemolytic uremic syndrome (HUS) (*1*). STEC constitute a heterogeneous group of bacteria abundant in the reservoir and in the environment (*2*). Transmission routes for human STEC infection are numerous and include contact with animal excreta, person-to-person transmission, and inadvertent ingestion of contaminated food and water. Many STEC serotypes have been recovered from humans (*3*,*4*). Among them, STEC O91 is the most common serogroup isolated from adult patients in Germany (*5*,*6*). The strains within this serogroup appear to be transmitted predominantly by food, because 1) food vehicles have been identified as the only risk factors for adults with sporadic STEC O91 infection in Germany (*6*); 2) O91 is the second most frequently isolated STEC serogroup in routine food samples (*5*); and 3) O91 is the only major STEC serogroup with no association between incidence of human infection and cattle density (*7*).

Whereas most human disease STEC serogroups possess, in addition to Shiga toxin, the *eae* gene encoding the adhesin intimin (*3*,*4*,*8*), STEC O91 consistently lack this virulence determinant (*8*,*9*). Despite frequent isolation of STEC O91 from humans, the clonal relatedness of the serotypes of this serogoup is poorly understood. Therefore, we investigated 100 human STEC O91 isolates to determine the clonal structure of STEC O91 and its association with disease.

## The Study

A total of 100 STEC O91 isolates were obtained from 1997 through 2007 from patients with HUS (n = 4), bloody diarrhea (n = 8), watery diarrhea without visible blood (n = 79), abdominal cramps without diarrhea (n = 1), or from asymptomatic carriers (n = 8); samples were from Germany (n = 96), Austria (n = 2; Austrian Reference Library, Innsbruck, Austria), Finland (n = 1; The National Public Health Institute, Helsinki, Finland), and Canada (n = 1; Public Health Agency of Canada, Guelph, Ontario, Canada). The 96 German O91 strains were recovered at the Institute of Hygiene, University of Münster, Münster, and the Robert Koch Institute, Wernigerode, Germany. The strains included all human isolates of this serogroup that were recovered during the study period in Germany and for which complete clinical information was available. The strains correspond to all O91 serotypes associated with human diseases from sporadic cases in Germany in that interval. Thirty-five strains have been described previously (*4*,*8*,*10*).

The age of patients from whom the STEC O91strains originated ranged from 4 months to 89 years (median 28 years, interquartile range 12–38 years). The most severe symptom was recorded for each patient. Diarrhea was defined as ≥3 semisolid or liquid stools per day. Bloody diarrhea was defined as diarrheal stools containing blood visible to the naked eye. HUS was defined as a case of microangiopathic hemolytic anemia (hematocrit <30% with peripheral evidence of intravascular hemolysis), thrombocytopenia (platelet count <150,000/mm^3^), and renal insufficiency (serum creatinine concentration greater than the upper limit of normal for age) (*11*). Asymptomatic carriers were apparently healthy persons without diarrhea; their stools were submitted as noted above.

Strains were isolated using Shiga toxin–encoding genes as diagnostic targets (*12*) and then serotyped phenotypically (*13*). All strains were verified as O91 by using PCR targeting *wzy*_O91_, a component of the *rfb* gene cluster that synthesizes the O91 antigen (*14*). Multilocus sequence typing (MLST) and phylogenetic analysis were performed as described (*4*). All allelic sequences were deposited in the *E. coli* MLST database (http://mlst.ucc.ie/mlst/dbs/Ecoli). The minimum spanning tree was generated from all 100 O91 sequence types (STs) and compared with the HUS-associated enterohemorrhagic *E. coli* (HUSEC) collection (*4*) to display the distribution of the STs compared with all known STs associated with HUS.

We tabulated disease severity according to STs. To study the relationship between ST442 and disease severity, patients were categorized into those with HUS, those with bloody diarrhea, and, serving as reference group, those with nonbloody diarrhea or those that asymptomatically excreted these organisms. Univariate associations were computed by using exact logistic regression. p values <0.05 were considered statistically significant. STATA release 10.0 (StataCorp LP, College Station, TX, USA) was used for statistical analysis.

The 100 STEC O91 strains resulted in 10 different STs. Of these, STs 33 and 442 were most common (63 and 20 isolates, respectively). Six additional STs (690, 1048, 1051, 1052, 1053, 1054) were single-locus variants of ST33, indicating their close relationship. The 2 remaining STs (641 and 1020) were not closely related to any other ST of the serogroup O91 strains. Detailed analysis of the 7 housekeeping genes used for MLST demonstrated the *fumC* gene sequence alone could differentiate 5 STs of the O91 strains. The comparison of all O91 STs with all STEC STs and serotypes associated with HUS (HUSEC collection) is displayed in the Figure.

**Figure:**
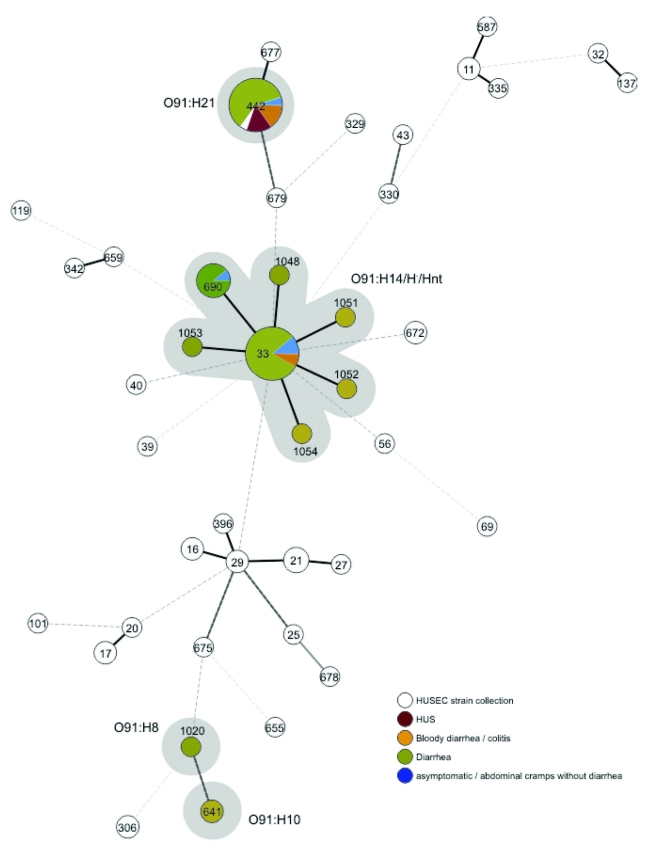
Minimum spanning tree based on the multilocus sequence typing allelic profiles portraying the clonal distribution of the 100 *Escherichia coli* O91:H8/H10/H14/H21/H^–^/Hnt isolates (highlighted in gray) associated with different diseases in relation to the hemolytic uremic syndrome–associated enterohemorrhagic *E. coli* collection. Each dot represents a given sequence type, and the size of each circle is proportional to the number of strains analyzed. Connecting lines show the number of identical alleles between 2 STs (thick black line, 6 of 7 alleles identical; thick gray line, 5 alleles identical; thick dashed line, 4 alleles identical; thin dashed lines of increasing length, <3 alleles identical).

H antigens associated with O91 were H8 (n = 1), H10 (n = 2), H14 (n = 52), H21 (n = 20), Hnt (H-antigen nontypeable) (n = 10), and H^–^ (H-antigen nonmotile) (n = 15). In serotype O91:H8, O91:H10, and O91:H21 strains, the STs were serotype-specific. However, ST33 and its single-locus variants represented all strains of serotypes O91:H14, O91: H^–^, and O91:Hnt.

A single sequence type, ST442, accounting for 20% of all strains, was found among each of the four O91 isolates from patients with HUS (Table 1), a highly significant association (odds ratio [OR] 27.8, 95% confidence interval [CI] 3.3–∞, p<0.01; Table 2). ST442 strains were also more frequently isolated from patients with bloody diarrhea than were strains belonging to other STs (3/20 [15.0%] vs. 5/80 [6.3%] respectively), but this difference was not statistically significant (Table 2). The overall association with severe disease, defined as either HUS or bloody diarrhea, was strong (OR 7.8, 95% CI 1.83–36.6, p<0.01). Severe illness was noted for 7 (35.0%) of 20 patients infected by ST442 strains, but only for 5 (6.3%) of 80 patients infected by STEC O91 of other STs (Table 2). Patients with bloody diarrhea were younger (median age 12 years) than patients who had mild or no symptoms (median age 20 years). However, this difference was not observed for the 4 patients with HUS (median age 21 years); in this instance, 2 were adults, 1 was 39 months old, and 1 was unknown.

**Table 1:** Distribution of disease severity across 10 different sequence types of 100 STEC O91 strains isolated from humans*

ST (Serotype)	Most severe symptom of patients infected with STEC O91				Total no. strains
	HUS (n = 4)	BD (n = 8)	D (n = 79)	A (n = 9)	
ST33 (O91:H14/H^–^/Hnt)	0	5	51	7	63
ST442 (O91:H21)	4	3	12	1†	20
ST641 (O91:H10)	0	0	2	0	2
ST690 (O91:H14)	0	0	8	1	9
ST1020 (O91:H8)	0	0	1	0	1
ST1048 (O91:H14)	0	0	1	0	1
ST1051 (O91:H14)	0	0	1	0	1
ST1052 (O91:H14)	0	0	1	0	1
ST1053 (O91:H14)	0	0	1	0	1
ST1054 (O91:Hnt)	0	0	1	0	1

*ST, sequence type; STEC, Shiga toxin–producing *Escherichia coli*; H, H-antigen nonmotile strains; Hnt, H-antigen nontypeable strains; HUS, hemolytic uremic syndrome; BD, bloody diarrhea; D, diarrhea without visible blood; A, asymptomatic infection (1 patient infected with ST442 [STEC O91:H21] had abdominal cramps without diarrhea). †Abdominal cramps without diarrhea.

**Table 2:** Univariate associations of STEC O91 of sequence type 442 with severe disease by use of exact logistic regression*

Severe disease	ST442, no. (%)	Non-ST442, no. (%)	Odds ratio	95% Confidence interval	p value
HUS	4 (20)	0 (0)	27.8†	3.29–∞	<0.01
BD	3 (15)	5 (6)	3.4	0.47–20.1	0.25
HUS or BD	7 (35)	5 (6)	7.8	1.83–36.6	<0.01

*Reference group consisted of persons who had nonbloody diarrhea or who were asymptomatic carriers. STEC, Shiga toxin-producing *Escherichia coli*; ST, sequence type; HUS, hemolytic uremic syndrome; BD, bloody diarrhea. †Mean unbiased estimate.

## Conclusions

To gain insight into the clonal structure of STEC O91, we determined the relatedness of 100 strains isolated from patients and correlated the clonal lineage to the clinical outcome of the infection. MLST analysis divided the O91 isolates into 10 different STs, whereas classical serotyping identified only 4 complete serotypes (O- and H-antigen). Moreover, MLST was able to type all 25 nonmotile (H^–^) or nontypeable (Hnt) O91 strains. The analysis demonstrated that the *fumC* gene from the 7 genes used for MLST was the most heterogeneous and enabled strain differentiation into 5 different STs, among these ST442. It might therefore be a candidate for first-line single-locus sequence typing.

HUS or bloody diarrhea without HUS was significantly associated with ST442, which was represented by serotype O91:H21 only. However, Pradel et al. also reported a case of HUS associated with an O91:H10 isolate that could be differentiated from O91:H21 by using ribotyping (*15*). In our study, known virulence determinants such as cytolethal distending toxin V or Shiga toxin 2d activatable by elastase in O91:H21 strains (*8*,*10*) might contribute to the higher virulence of O91:H21 (ST442). However, further studies of the mechanisms behind the emergence of ST442 in Germany and additional analysis of global O91 isolates are needed. With the MLST approach described, trends and changes in STEC O91 epidemiology and human infections can be carefully surveyed.
